# Human jugular vein collapse in the upright posture: implications for postural intracranial pressure regulation

**DOI:** 10.1186/s12987-017-0065-2

**Published:** 2017-06-17

**Authors:** Petter Holmlund, Elias Johansson, Sara Qvarlander, Anders Wåhlin, Khalid Ambarki, Lars-Owe D. Koskinen, Jan Malm, Anders Eklund

**Affiliations:** 1 Department of Radiation Sciences, Umeå University, 901 87 Umeå, Sweden; 2 Department of Pharmacology and Clinical Neuroscience, Umeå University, 901 87 Umeå, Sweden; 3 Umeå Centre for Functional Brain Imaging, Umeå University, 901 87 Umeå, Sweden

**Keywords:** Jugular vein, Collapse, Intracranial pressure, Posture, Physiology

## Abstract

**Background:**

Intracranial pressure (ICP) is directly related to cranial dural venous pressure (*P*_*dural*_). In the upright posture, *P*_*dural*_ is affected by the collapse of the internal jugular veins (IJVs) but this regulation of the venous pressure has not been fully understood. A potential biomechanical description of this regulation involves a transmission of surrounding atmospheric pressure to the internal venous pressure of the collapsed IJVs. This can be accomplished if hydrostatic effects are cancelled by the viscous losses in these collapsed veins, resulting in specific IJV cross-sectional areas that can be predicted from flow velocity and vessel inclination.

**Methods:**

We evaluated this potential mechanism in vivo by comparing predicted area to measured IJV area in healthy subjects. Seventeen healthy volunteers (age 45 ± 9 years) were examined using ultrasound to assess IJV area and flow velocity. Ultrasound measurements were performed in supine and sitting positions.

**Results:**

IJV area was 94.5 mm^2^ in supine and decreased to 6.5 ± 5.1 mm^2^ in sitting position, which agreed with the predicted IJV area of 8.7 ± 5.2 mm^2^ (equivalence limit ±5 mm^2^, one-sided t tests, p = 0.03, 33 IJVs).

**Conclusions:**

The agreement between predicted and measured IJV area in sitting supports the occurrence of a hydrostatic-viscous pressure balance in the IJVs, which would result in a constant pressure segment in these collapsed veins, corresponding to a zero transmural pressure. This balance could thus serve as the mechanism by which collapse of the IJVs regulates *P*_*dural*_ and consequently ICP in the upright posture.

**Electronic supplementary material:**

The online version of this article (doi:10.1186/s12987-017-0065-2) contains supplementary material, which is available to authorized users.

## Background

Cerebral venous pressure and intracranial pressure (ICP) varies with body posture [1–5]. Since ICP has mostly been studied in the supine position, little is known about the underlying mechanisms controlling these variations. Increased knowledge of the mechanisms that regulate how ICP changes with posture may aid in understanding the pathophysiology and improving the treatment of diseases such as cerebral venous thrombosis [6], traumatic brain injury [7], idiopathic intracranial hypertension (IIH) [8] and hydrocephalus [9]. Furthermore it has the potential to contribute to the design of new cerebrospinal fluid (CSF) shunts that better prevent over drainage when patients are upright [10].

In a recent study [5], we proposed a model where ICP in the upright posture is explained by a hydrostatic pressure reference point for the venous system at the level of the neck, and we suggested that this pressure reference point is related to the collapse of the internal jugular veins (IJVs). Furthermore, a recent theoretical analysis of CSF compliance has also indicated that IJV collapse likely plays an important role in the CSF dynamics in the upright human [11]. However, neither of these studies investigated how the suggested neck-level pressure reference point is formed and upheld by the well-known jugular venous collapse. This motivates further studies of IJV collapse and how it translates to an effect on ICP.

The link between equilibrium ICP and venous pressure is described by Davson’s equation [12–14] for CSF absorption:

1 $$ICP = R_{out} \,I_{form} + P_{dural}$$

where *R*_*out*_ is the CSF outflow resistance, *I*_*form*_ the formation rate of CSF and *P*_*dural*_ the pressure in the dural veins. Equation 1 postulates that a change in venous pressure (and thus *P*_*dural*_) should be followed by a corresponding change in ICP. It is known that venous pressure in the upper body decreases due to hydrostatic effects in the upright posture [15], with a venous hydrostatic indifference point slightly below the level of the heart [16], but due to collapse of the IJVs the cranial venous pressure is not as negative in the upright posture as these hydrostatic effects would suggest [17]. While the IJVs collapse in upright, in general they do not totally occlude in this position [18–20], which means that the fluid communication between the heart and brain is not disrupted; rather the collapse likely affects the IJV pressure more like a Starling resistor.

In this study, we evaluated a biomechanical description of the collapsing IJVs that could explain previous observations of upright ICP [3, 5] through a segment of zero transmural pressure in the neck. The description is based on the idea that the highly flexible IJVs adjust their shape to allow for transmission of the surrounding atmospheric pressure to the internal venous pressure of these collapsed vessels. This behaviour has been observed in experimental bench studies of collapsible rubber tubes inclined to some angle [21–24] and should be applicable in vivo if the IJVs are sufficiently flexible, i.e. wall forces are negligible, when in the collapsed state. Such a physiological mechanism should result in specific cross-sectional areas that are uniquely predicted by the IJV flow rate and body posture. For validation, we measured the IJV cross-sectional area and flow velocity in healthy volunteers using ultrasound. An agreement between measured and predicted IJV area in the upright posture would support the hypothesized description of IJV collapse and how venous collapse can allow for a venous pressure reference point at neck level in the upright human.

## Methods

In summary, a theoretical expression for the collapsed IJV cross-sectional area was derived based on the assumption of zero transmural pressure along the collapsed venous segment. This theoretical description was then evaluated by comparing the predicted IJV area with IJV area measurements in healthy volunteers.

### Theoretical expression for the cross-sectional area

**Fig. 1:**
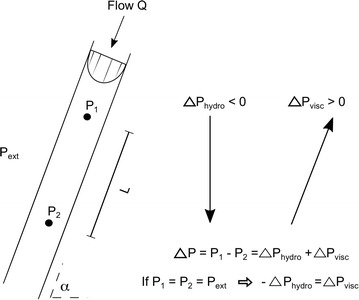
Description of pressure in a collapsed vessel. The description is based on the assumption of zero transmural pressure in the collapsed jugular vein. Thus, to achieve zero transmural pressure in the collapsed section of the vessel the *internal* pressures at any two levels 1 and 2 must be equal to the external pressure, i.e. *P*_*1*_ = *P*_*2*_ = *P*_*ext*_. This means that internal pressure cannot change along the vein from level 1 to level 2 and the pressure (hydrostatic and viscous) components must cancel each other in this segment. The *arrows* indicate the direction of increasing pressure for the two pressure components inside the vessel. *L* is the distance between the two points in question and *α* is the tilt angle of the vessel. Since near-zero (i.e. near atmospheric) pressures are expected around the IJVs [24, 28, 29], the internal pressure should also be near-zero after collapse

For zero transmural pressure to hold along the collapsed IJVs at neck-level, assuming a constant surrounding pressure, the pressure at any two points along the collapsed segment must be the same and there will be no *pressure difference* between these two points (these principles are illustrated in Fig. 1). The pressure difference between two points in an inclined vessel is due to two major pressure contributions: the hydrostatic pressure of the blood column and the viscous losses due to flow resistance [22]. The hydrostatic pressure difference between two points of interest in a fluid column is described by:

2 $$\Delta {P}_{hydro} = \;\rho\;{g}\;{h}\; = \;\rho\;{g\;L\; \text{sin}}\alpha$$

where *h* is the height of the fluid column, *L* the total distance between the points of interest, *α* the tilt angle, *g* the gravitational acceleration and *ρ* the fluid density (Fig. 1). The viscous losses in a vein can be estimated by the modified Hagen–Poiseuille equation for elliptical tubes [21]:

3 $$\Delta {P}_{visc} = {RQ}\; = {k}\frac{{8\;\uppi \;\mu \;{L}}}{{{A}^{2} }}{Q}$$

where *R* is the flow resistance, *Q* the flow rate, *L* the total distance between the points of interest, *μ* the viscosity and *A* the cross-sectional area of the vein. The constant *k* = (*a* ^*2*^ + *b* ^*2*^)/2*ab* describes the shape of the vessel and is here called the ellipse factor; *a* and *b* are the semi-major and semi-minor axes of the ellipse [21]. For a circular tube, *k* = *1* and Eq. 3 then corresponds to the standard Hagen–Poiseuille equation. The rationale for the inclusion of the ellipse factor is to account for how the non-circular/semi elliptical shape of the collapsing IJVs (see Additional file 1: Figure S1) affects the flow resistance.

Then, equating Eqs. 2 and 3, gives us an expression for the IJV cross-sectional area (*A*_*c*_) (Fig. 1) as a function of tilt angle *α* and maximum flow velocity *U*_*max*_:

4 $${A}_{c} = \frac{{ 4\;\uppi \;\mu \;{k\; U}_{max} }}{{\rho\; {g\; \text{sin}}\alpha }}$$

where we made use of *Q* = *AU*_*max*_/2, assuming a parabolic flow profile (applicable for both circular and elliptic cross-sections [25]). Equation 4 is the key relationship for the suggested IJV collapse mechanism and the relationship evaluated in this study. It is important to emphasize that the relationship is only meant to describe the collapsed state of a vessel. For positive transmural pressures, Eq. 4 should cease to be valid and IJV area should instead exceed *A*_*c*_, e.g. in supine and at low upper body tilt angles where the hydrostatic column from the heart is small and the IJVs are inflated by the positive central venous pressure.

### Subjects

The study included 17 healthy volunteers (10 women) of age 45 ± 9 years (mean ± SD). Volunteers were recruited via an advertisement in a local newspaper and were considered eligible if they were without any past or present neurological, cardiovascular or psychiatric diseases. In addition, they had to have normal blood pressure (<140/90) and be within the age range 30–60 years. Subjects using any medication affecting the cardiovascular system or central nervous system were excluded.

### Ultrasound measurement protocol

To study the validity of Eq. 4, we measured jugular cross-sectional area, ellipse factor *k* and maximum velocity *U*_*max*_ with ultrasound. The jugular veins were examined using a GE Vivid E9 ultrasound system with a 9L linear probe (4–8 MHz) (General Electric Healthcare, Chicago, IL, USA). Brightness-mode was used for investigating the cross-sectional area of the IJVs at three different neck levels, on both sides (located 23 ± 2, 27 ± 3 and 29 ± 3 cm from the bottom of the sternum, respectively). The rationale for using three levels was to identify the segment with the smallest area in sitting posture, in order to ensure that the cross-sectional area was investigated in the collapsed region of the vein. This segment was then used in the analysis of agreement between measured and predicted IJV area.

The subjects were placed on their backs on a bed with an adjustable backrest and the ultrasound measurements were performed with the upper body/backrest at tilt angles of 0° (supine), 16° (half-sitting) and 71° (sitting). The half-sitting position was included to test Eq. 4 as a potential tool for determining the occurrence of collapse, as this angle is close to where our previous study suggested that venous collapse may start to influence ICP [5]. The angles were measured using a digital inclinometer (mini digital protractor) placed on the backrest of the bed. The leg rest was kept horizontal at all times. Each level was maintained for at least 8 min, during which the measurements took place. To avoid any local increase in the external pressure, the ultrasound probe was held so that only the ultrasound gel was in contact with the skin, i.e. the probe and the skin were only held together by the surface tension of the gel, as indicated by a lack of signal at the edges of the ultrasound images. An ultrasound sequence was saved for each measurement, consisting of 89–90 frames and a time span of roughly 3 s.

### Measuring the IJV cross-sectional area

In the ultrasound images, a region of interest (ROI) was manually drawn along the circumference of the jugular vein, and the cross-sectional area was calculated as the area within the ROI (see Additional file 1: Figure S1). We assessed the absolute minimum and maximum for each sequence and the average of the two was used as an estimate of the mean area for each sequence (*A*_*meas*_). In addition to the cross-sectional area, the major and minor axes of the IJVs were also measured (in the same frames as the minimum and maximum area), in order to get an estimate of the ellipse factor (Additional file 1: Figure S1). As was the case with the cross-sectional area, the mean ellipse factor for each sequence was estimated as the average of the two ellipse factors. MATLAB (version R2012b, The Mathworks, Natick, MA) was utilized for all calculations.

### Ultrasound blood flow velocity measurements

The blood flow velocity was measured directly after the three measurements of cross-sectional area, at the same three neck level locations. The velocity was estimated using angle-corrected pulsed-wave ultrasound. The time average velocity was calculated for each ultrasound sequence by manual analysis of the Doppler data. Velocity was measured at the centre of the vessel, with the assumption that we thus measured the maximum velocity in the vessel (*U*_*max*_). The velocity analysis was performed using MATLAB. One vessel with no detectable blood flow (i.e. fully occluded somewhere) was excluded from the analysis, since the assumptions for the hydrostatic-viscous pressure balance (Fig. 1) would not be valid if flow was zero. This resulted in a total of 33 IJVs for the analysis.

### Predicting IJV collapse area

The main analysis consisted of comparing the predicted IJV collapse area *A*_*c*_ (according to Eq. 4) with the measured mean IJV area *A*_*meas*_. Constants were set to *ρ*_*blood*_ = 1060 kg/m^3^, *μ*_*blood*_ = 3.8 × 10^−3^ Pa s and *g* = 9.81 m/s^2^. The IJV level used for the analysis was the one with the smallest area in sitting position: level 1 for 21 IJVs and level 2 for 12 IJVs. To calculate *A*_*c*_ individual ellipse factors *k*, individual flow velocities and tilt angles of the backrest were inserted in Eq. 4.

### Statistics

A test of equivalence [26] was used for comparing the predicted and measured IJV area in sitting position. The equivalence limits for the difference were set to ±5 mm^2^, and two one-sided t tests (TOST) were performed, one for each limit, with the alternative hypothesis representing equivalence. The equivalence limit was based on error estimations assuming a measurement inaccuracy and physiological variability (e.g. respiratory and autoregulatory effects) of 25% in *U*_*max*_, *k* and *A*_*meas*_, and an IJV area in upright of around 10 mm^2^ [20, 27]. The significance level was set to p < 0.05. All statistical calculations were performed using built-in MATLAB functions. Measurement results are presented as mean ± SD unless otherwise specified.

## Results

**Fig. 2:**
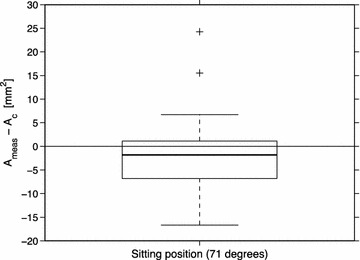
Boxplot of the comparison between predicted and measured IJV cross-sectional area. The *within-box line* represents the median and *the box* shows the first and third quartiles. The *whiskers* show maximum and minimum while outliers are represented by *plus signs*. Median = −1.8 mm^2^

In sitting, the IJV cross-sectional area predicted by the pressure balance (Eq. 4) was found to be equivalent to the measured cross-sectional area (*A*_*meas*_ = 6.5 ± 5.1 mm^2^ and *A*_*c*_ = 8.7 ± 5.2 mm^2^, equivalence test: limits ±5 mm^2^, p = 0.03). This was further supported by a paired t test, which showed no significant difference between *A*_*meas*_ and *A*_*c*_ (p = 0.14). A boxplot of the difference between *A*_*meas*_ and *A*_*c*_ is shown in Fig. 2. The analysis of the half-sitting position showed that 27% of the 33 IJVs had an *A*_*meas*_ within the equivalence limits of *A*_*c*_ ± 5 mm^2^ or below *A*_*c*_ (using individual values of *A*_*c*_) indicating that in these cases collapse had occurred already at this tilt angle.

*A*_*meas*_ was 94.5 ± 53.3, 40.1 ± 33.8 and 6.5 ± 5.1 mm^2^ in supine, half-sitting and sitting position, respectively. The change in area going from supine to sitting was thus 88.0 ± 53.1 mm^2^ (one-sided paired t test, p < 0.01). The area while sitting was 11 ± 14% of the supine value. The average maximum and minimum *A*_*meas*_ observed in sitting position were 8.1 and 5.0 mm^2^, respectively. The ellipse factor *k* was 1.2 ± 0.3, 1.6 ± 0.7 and 2.1 ± 1.0, illustrating a change in IJV shape from close to circular in supine to more elliptical in sitting position. The maximum velocity *U*_*max*_ increased from 18 ± 19 cm/s in supine to 38 ± 34 cm/s in half-sitting and 89 ± 39 cm/s in sitting position. In one IJV, an extremely high value for *U*_*max*_ was observed in supine position. Without this value, the supine *U*_*max*_ would have been 16 ± 10 cm/s.

## Discussion

This study investigated the collapse of the IJVs in healthy subjects when going from supine to sitting. The results showed that measured IJV area and the area predicted by hydrostatic-viscous pressure balance agreed well in upright/sitting position, which supports zero transmural pressure in the collapsed IJVs in the upright human. The pressure balance could thus serve as the mechanism by which collapse of the IJVs regulate ICP in the upright posture to the levels reported in ICP studies.

Our aim in this study was to investigate how the IJV collapse could explain the previously observed changes in ICP [3, 5] when going from supine to sitting position. A hydrostatic-viscous pressure balance in the collapsed IJVs would introduce a segment where pressure remains constant, and the top of this segment would then serve as a new pressure reference point for cranial venous pressure. Since the driving force behind the pressure balance is a zero transmural pressure, the surrounding tissue pressure should determine the actual internal venous pressure and thus the pressure at this reference point. Previous studies of pressure around the IJVs in the neck have indicated that the surrounding pressure may range from slightly negative [28] to slightly positive [24, 29], but still close to zero (i.e. atmospheric pressure). This would be in agreement with studies of internal IJV pressure, where the pressure has ranged from zero to slightly positive in the collapsed IJVs [15, 30]. Such magnitudes of the surrounding pressure would also explain the ICP changes observed previously [5].

*P*_*dural*_, and subsequently ICP, will depend on the position of the top of the collapse. Inter-individual differences in collapse length can thus yield inter-individual variations in *P*_*dural*_ and ICP. A collapse at neck level would result in a negative *P*_*dural*_ corresponding to a hydrostatic column of around 10 cm and an ICP that is close to zero or slightly negative, which is in agreement with previous ICP observations [3, 5].

The results of this study further support the idea of IJV collapse as an active part in the regulation of cranial venous pressure and thus also ICP. Since humans spend most of their day in an upright position, alterations in the IJV collapse function might be of importance in diseases with a suspected disturbance of the ICP dynamics, e.g. hydrocephalus, IIH and postural headache. Furthermore, since both area and flow estimations can quickly be performed using ultrasound, determination of the angle where collapse occurs could contribute to treatment of traumatic brain injury, where slight head-of-bed tilt is used to lower ICP [31, 32] and the optimal degree of head elevation is a matter of debate [33, 34]. Understanding how gravity regulates ICP is also of utmost importance for understanding changes in ICP when gravity is removed, i.e. in microgravity. One case of particular interest is the visual impairment syndrome seen in astronauts on long-duration space missions [35, 36] where an ICP disturbance is believed to be the main cause [37].

With a confirmed zero transmural pressure in collapsed IJVs, the simple expression for *A*_*c*_ (Eq. 4) could be used to identify a collapsed IJV. This approach could then easily be applied in a clinical setting when investigating cranial venous pressure and ICP, since the IJVs are superficial veins that are easy to assess with ultrasound. Our measurement results in this study indicated that about one-fourth of the IJVs (27%) had already collapsed in the half-sitting position, verifying an inter-individual variation in the angle where collapse first occurs. The reason for the differences in collapse angle should mainly be inter-individual variations in central venous pressure and height.

**Fig. 3:**
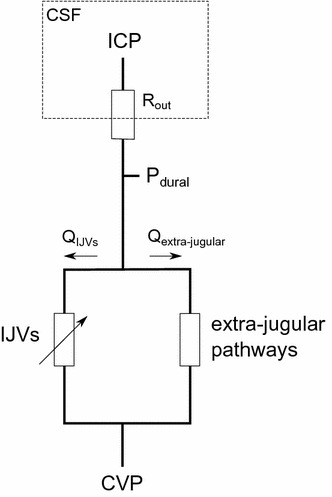
A circuit analogue of the cerebral venous drainage and the CSF absorption, which illustrates the relation between venous pressure and ICP. The IJVs form a parallel circuit with the extra-jugular pathways (e.g. the vertebral venous plexus). The figure illustrates how dural venous pressure (*P*_*dural*_) can be determined by following either of the possible venous pathways, and highlights the variable resistance of the collapsible IJVs. Q_IJV_: IJV blood flow, Q_extra-jugular_: extra-jugular blood flow

In this study we utilize the jugular venous pathway to understand the cranial venous pressure. Previous studies have successfully modelled the cranial venous drainage, including the IJVs, as a parallel system analogous to an electrical circuit in order to predict blood flow distributions [38, 39]. In such a system, all possible venous pathways from the brain to the heart affect the distribution of blood flow. However, when interested specifically in the pressure dynamics, any single pathway can be analysed, since all of them must yield the same central venous pressure and cranial venous pressure regardless of the pathway chosen (Fig. 3). This supports our use of the single pathway through the IJVs, as this provides sufficient information as long as the IJVs are not totally occluded (in that case, fluid communication would be broken in the IJVs and cranial venous pressure would be regulated by other, non-occluded pathways, such as the vertebral veins [19, 38, 40]).

While equivalence of measured and predicted area was strongly supported by the results, the observed difference of 2.2 mm^2^ still opens the possibility that *A*_*meas*_ is slightly smaller than *A*_*c*_, meaning that the viscous losses could be larger than the hydrostatic component, leading to a pressure increase along the collapsed jugulars (from the proximal to the distal end). For example, if the equivalence limit were decreased to 3 mm^2^, the two areas would not be considered equivalent (p = 0.29). However, since the difference is well within reasonable limits for measurement accuracy, this result could simply be due to systematic errors in the measurements.

The results in Fig. 2 reveal deviations from the predicted area in some specific cases. For the IJVs corresponding to the two extreme cases, it was the measured area that was unexpectedly large in the upright posture (*A*_*meas*_ was 25.1 and 19.7 mm^2^, respectively). Therefore, we believe it is possible that our three measurement sites did not successfully capture the collapse of these two IJVs during the ultrasound examination. The IJVs corresponding to the largest overestimation of the predicted area could possibly be explained by overestimation of the ellipse factor, as these IJVs had comparatively high ellipse factors (i.e. a very flattened shape) and since these measurements were sometimes difficult to perform for the smallest IJVs. However, it is also possible that the use of the correction by the ellipse factor was insufficient for these cases, i.e. that this assumption had reached its limit, and that the viscous losses were no longer well described by the modified Hagen–Poiseuille equation (Eq. 3). For example, if the IJVs with an ellipse factor *k* > 2.5 were excluded, the mean difference between *A*_*meas*_ and *A*_*c*_ was only 0.5 mm^2^ (number of IJVs = 27). Thus, suggesting that highly flattened IJVs require a modified expression for the factor *k*, which is implicated by experimental studies of highly collapsed tubes [41].

In addition to the measurement uncertainties in ultrasound area and velocity measurements, the variability between predicted and measured IJV area (Fig. 2) could be due to variation in tissue pressure or in vessel rigidity along the IJVs. This would result in an IJV area that is expected to vary along the vessel, with a magnitude fluctuating around the predicted area. Furthermore, while we believe that the inertial effects, e.g. from variations in flow [42, 43], are relatively small in the upright IJVs, inertial effects are present [44] and can be a factor contributing to the observed differences, thus this is an area of improvement where further analysis would be interesting. In spite of these limitations, since the predicted area in the upright posture was in good agreement with the measured area on group level, the results support the hypothesized physiological mechanism, although we acknowledge that the limitations of the above assumptions need to be addressed before individual area predictions can be fully implemented.

## Conclusions

In conclusion, the agreement between predicted and measured IJV cross-sectional area in sitting indicates that hydrostatic effects are indeed cancelled by the viscous losses in the collapsed IJVs, which supports the occurrence of a zero transmural pressure segment in the IJVs in the upright human. The hydrostatic-viscous pressure balance could thus serve as the mechanism by which collapse of the IJVs regulates ICP in the upright posture.

## Additional file

**Additional file 1: Figure S1.** Illustration of how IJV cross-sectional area (area within manually drawn circumference) was assessed and the ellipse major and minor axes (straight lines) were estimated in the ultrasound images. The upper image shows a measurement in supine and the lower a measurement at 16° upper body tilt (half-sitting).

## Abbreviations

CSF: cerebrospinal fluid
CVP: central venous pressure
ICP: intracranial pressure
IIH: idiopathic intracranial hypertension
IJV: internal jugular vein
ROI: region of interest
*P*_*dural*_: dural venous pressure
*U*_*max*_: maximum flow velocity
*A*_*meas*_: measured jugular vein cross-sectional area
*A*_*c*_: predicted jugular vein cross-sectional area
*k*: estimated ellipse factor
